# Impact of a Professional Nutrition Program on a Female Cross Country Collegiate Athlete: A Case Report

**DOI:** 10.3390/sports6030082

**Published:** 2018-08-19

**Authors:** Majid Mufaqam Syed-Abdul, Dhwani Satishkumar Soni, Jason Daniel Wagganer

**Affiliations:** Departments of Health, Human Performance, and Recreation, Southeast Missouri State University, Cape Girardeau, MO 63701, USA; dhwani_soni4890@yahoo.in (D.S.S.); jwagganer@semo.edu (J.D.W.)

**Keywords:** female athlete triad, energy balance, nutrition, track and field athlete

## Abstract

Low caloric intake or excessive energy expenditure can lead to a negative energy balance, which, in female athletes, may result in a condition called the female athlete triad. While several guidelines identified proper nutrition as a first line of treatment, little research has been reported to show the effect of a professional nutrition program (PNP) on the female athlete triad. The purpose of this case report was to measure the short- and long-term effects of a PNP on a female athlete presenting triad characteristics. A 20-year-old female track-and-field athlete at a Division I university who was in negative energy balance and amenorrheic underwent a one-month PNP. Short- and long-term effects measured by a dual X-ray absorptiometry scan prior to and after attending a PNP showed increased total energy intake from 2188 kcals to 3187 kcals, which resulted in an increase in body fat percent (BF%) from 4.7% to 6.7%. However, by the end of four months, energy intake and BF% (5.7% and 6.0%) values were reduced, respectively. After the twelve-month follow-up, BF% was increased (10.5%), suggesting that increasing energy intake to meet energy demands, without compromising athletic training, can be an effective treatment for the female athlete triad.

## 1. Introduction

Energy balance (i.e., energy intake equals energy expenditure) is a key factor in maintaining an athlete’s health. Low caloric intake or excessive energy expenditure (i.e., exercise training) can lead to a negative energy balance, which, in females, may result in a condition called the female athlete triad [1,2]. Prevalence of the female athlete triad has been increasing, at least partially, due to more female athletic participation [3]. The increased nutritional needs of a sport combined with psychological factors (i.e., the desire to look thin) may lead to low nutrient intake without an eating disorder [4,5], menstrual disturbances [6], and/or low bone mineral density (BMD) [1,2,7]. Therefore, it is important to educate athletes and coaches about warning signs and appropriate treatment options associated with the female athlete triad [3]. Several pharmacological and nonpharmacological therapies have been outlined for the treatment of the female athlete triad [1,3,6,8]. While pharmacological therapies are to be utilized by clinicians under extreme conditions, nonpharmacological treatments [i.e., professional nutrition programs (PNP)] are currently implemented first [6]. However, limited literature is available on the effect of energy balance as a female athlete triad treatment. Therefore, the purpose of this case report was to measure the effects of balanced energy intake, through a PNP, on body fat percentage (BF%) and BMD in a female athlete with characteristics of the female athlete triad. The second purpose of this case report was to measure the short- and long-term effects of a PNP.

## 2. Materials and Methods

### 2.1. Case Report Design

**Figure 1:**
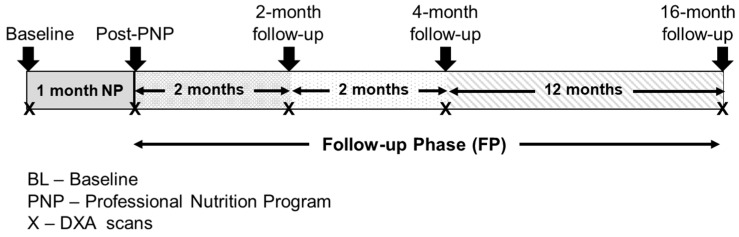
Case report timeline.

The 17-month case report was composed of a one-month PNP, and three post-PNP follow-ups (two, four, and sixteen months) (Figure 1). The participant maintained normal physical activity levels during the case report.

### 2.2. Recruitment (RC)

This case report included a Division I female track and field athlete who identified with two (energy deficit and menstrual disturbance) out of three characteristics of the female athlete triad and reported continuous weight loss. Prior to the case report, the participant signed a written informed consent, which was approved by the Institutional Review Board and fully outlined the purpose, protocols, procedures, and risk associated.

### 2.3. Nutrition Program and Dietary Records

The participant selected the off-campus one-month PNP, which was designed to provide guidance and education on proper nutrition intake based upon lifestyle. As the goal of the program was to increase overall body weight and BF%, total calorie intake, including total amount of fat and carbohydrates, was increased during the program (Table 2). The program was supervised by a registered dietitian and the participant’s coach. After the one-month PNP, the participant was encouraged to independently continue the dietary advice for the next sixteen months. The participant recorded daily dietary intake via a professional website (^©^2017 MyNetDiary Inc., Cherry Hill, NJ, USA), and physical activity during and after the completion of the PNP. The 24-hour dietary intake record and physical activity data was collected for one week prior to baseline, at the completion of PNP (post-PNP), and at the two-month and four-month follow-up. However, because of the cost associated with the website and participant’s compliance, diet intake and physical activity data was not collected for the last twelve-month follow-up phase.

### 2.4. Measurements

Body weight and basal metabolic rate (BMR) were measured and calculated via the Harris–Benedict equation for females (utilizing the highly active category) [9] at all data collection time points. Body composition and BMD was measured by Dual-energy X-ray Absorptiometry (DXA) (Lunar Prodigy Advance PA+300123, GE Healthcare, Pewaukee, WI, USA). To measure the short- and long-term effects of PNP, DXA scans were performed during baseline, post-PNP, 2-month follow-up, 4-month follow-up, and 16-month follow-up. Participant’s menstrual cycles were not recorded during this case report, because her personal physician prescribed birth control prior to the initiation of this study. However, the female in this study reported being amenorrheic for >12 months prior to the case report data collection.

## 3. Results

**Figure 2:**
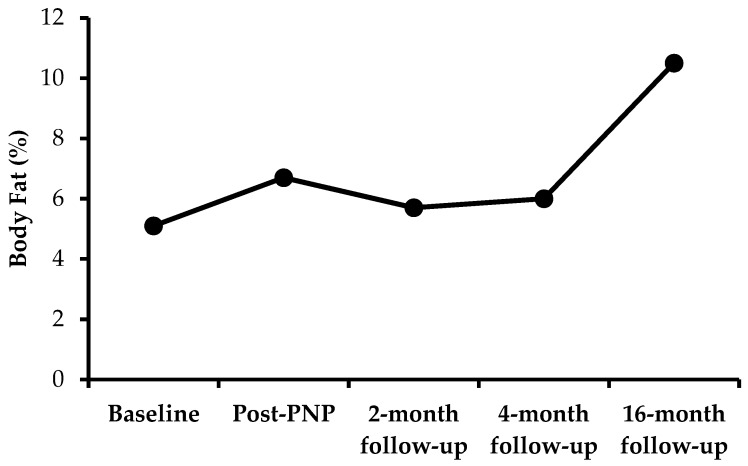
Body fat percent during and after the PNP.

Descriptive characteristics and dietary intake/exercise data are shown in Table 1 and Table 2. Compared with the baseline, an increase in total energy intake was observed at the post-PNP, two-month follow-up, and four-month follow-up. In support of these results, BMI was maintained, body weight was minimally increased at post-PNP and the 16-month follow-up, and an increase in fat mass and BF% was observed at the post-PNP time point (Table 1). Although total energy intake during the two-month follow-up (2796 kcal) and four-month follow-up (2781 kcal) were higher than the baseline (2188 kcal), dietary intake during the two-month follow-up and four-month follow-up were lower than the post-PNP time point (3187 kcal). Fat consumption was higher at the post-PNP time point. However, when compared with post-PNP (105 g), average fat consumption was reduced at the two-month follow-up (77 g) and four-month follow-up (74 g). Similarly, dietary fat (percent of total intake) was higher at the PNP time-point but was similar to baseline and the two-month and four-month follow-up. Total grams of carbohydrate and protein intake was also higher, and remained higher, at all time-points after the baseline. As shown in Figure 2, BF% was increased after completion of the program. However, this was reduced for up to four months and then, by the end of the 12-month follow-up, it was increased to 10.5%. Sugar intake increased during and after the PNP, whereas cholesterol intake did not change. Additionally, sodium intake increased during the PNP (5346 mg) and was lower at the two-month (3967 mg) and four-month follow-up (3914 mg). As instructed, the participant’s physical activity levels remained unchanged throughout the case report, however, total energy expenditure was reduced compared to the baseline (Table 2). Interestingly, the participant also reported improvements in well-being (eating more, felt good, and enjoyed weather) after completion of the program (PNP).

## 4. Discussion

The main purpose of this case report was to measure the effects of balanced energy intake, through a PNP, on BF% and BMD in a female athlete with characteristics of the female athlete triad. The energy intake for the participant in our case report, 2188 kcal/day at baseline, was similar to normal age- and BMI-matched healthy non-athlete females [5]. However, because of increased physical activity, our participant’s total energy requirements (3141 kcal/day) were higher than normal age- and BMI-matched non-athlete females (1707 kcal/day deficit) [10]. The PNP appeared to help the athlete more closely meet required energy demands (Table 2). However, the self-reported increase of 1000 calories/day at the post-PNP time-point, which produced a negligible 0.6 kg weight gain, was most likely inflated/inaccurate. It is possible that the increased caloric intake did better meet energy demands, but because of oral contraceptives preventing a menstrual cycle, it is not possible to definitively report whether the participant achieved an energy balance. It is also possible that the PNP stimulated the participant to self-report an increase in caloric intake as a mechanism to satisfy coaches and doctors. While pre-study caloric intake was not tracked, the participant reported consistent weight loss prior to the initiation of this study. Therefore, the PNP did stimulate the participant to increase caloric intake enough to stabilize body weight. The decreased energy intake at the two-month and four-month follow-up, along with decreased body fat mass, suggests the importance of constant monitoring by a PNP for body mass maintenance [6,8]. While some treatment therapies include reducing physical activity and utilizing pharmacological therapies to increase BF% [8], the results from our case report showed that body weight was maintained and an increase in energy intake improved BF% (Figure 2), which is consistent with previous research [11]. Moreover, these outcomes were achieved without compromising the training program, suggesting energy balance and/or an increase in caloric intake could be utilized as a primary means of treating the female athlete triad, rather than utilizing pharmacological therapies as discussed previously [6]. For the 16-month follow-up, daily food and exercise data were not recorded because of financial challenges, which was one of the limitations of our case report. While the American College of Sports Medicine (ACSM) position stand recommends that reduced physical activity and balanced energy intake will improve body composition [2], the International Olympic Committee (IOC) suggests the presence of energy balance alone may not be sufficient to treat the female athlete triad [7]. Moreover, because of the lack of past research and the lack of data collection in our case report at the 16-month follow-up, it is unclear of what individual or combined effects dietary intake and physical activity had on the improved BF% at the end of the twelve-month follow-up in our case report (Table 1). Interestingly, while running distance did not change, energy expenditure was reduced after completion of the program (post-PNP) and at the two-month and four-month follow-up. This is perhaps because of a decrease in exercise duration, suggesting improved mechanical efficiency of the participant, which may have resulted in less energy expenditure. Further, changes in BMD were not observed at the two-month and four-month follow-up, most likely because of the time it takes for changes to occur [12] and/or our participant was already greater than 1.0 g/cm^2^ (healthy adult levels) [13]. Additionally, no changes in BMD at the 16-month follow-up were observed, most likely because of the participant’s previous active lifestyle, which did not change during the timeline of this case report.

## 5. Conclusions

In summary, this case report showed that increased energy intake, to better meet the energy requirements, without compromising the exercise training program, increased BF% and maintained BMD after the PNP in a female athlete. Although the PNP minimally increased body weight, maintenance of BF% and BMD at the two-month and four-month follow-up, and increased BF% at the 16-month follow-up supported the short- and long-term benefits of the PNP for a female athlete who was experiencing consistent weight loss in the past. Although only one participant was followed in this case report, it is suggestive that monitoring an athlete’s nutrient intake (i.e., PNP) can be an effective means for treating characteristics of the female athlete triad.

## Figures and Tables

**Table 1:** Descriptive characteristics.

Characteristic	Baseline	Post-PNP	2-Month Follow-up	4-Month Follow-up	16-Month Follow-up
Age	20.6	20.7	20.9	21.1	22.1
Height (m)	1.75	1.75	1.75	1.75	1.75
Body weight (kg)	56.70	57.32	56.25	56.96	58.66
BMI (kg/m2)	18.46	18.66	18.31	18.55	19.10
BMR (kcal) 1	1428	1429	1418	1425	1436
Energy requirements (kcal) 1	3141	3143	3120	3134	3160
Fat mass (kg)	2.54	3.66	3.08	3.26	5.89
Fat (%BW)	4.7	6.7	5.7	6.0	10.5
Lean mass (kg)	51.61	51.12	50.67	51.16	50.31
Lean mass (%BW)	91.02	89.17	90.08	89.81	85.77
BMD (g/cm2)	1.188	1.197	1.206	1.169	1.192
BMC (g)	2561	2575	2560	2545	2520

Abbreviations: BMR—basal metabolic rate, BW—body weight, BMI—body mass index, BMD—bone mineral density, BMC—bone mineral content, PNP—professional nutritional program. ^1^ Basal metabolic rate and energy requirements were calculated using Harris–Benedict equation for females by using participant’s current BW and physical activity factor for highly active individuals.

**Table 2:** Average energy consumption and expenditure data.

**Energy Consumption/day**	**Baseline**	**Post-PNP**	**2-Month Follow-up**	**4-Month Follow-up**
Kilocalories consumed	2188	3187	2796	2781
Fat (g)	69	105	77	74
Fat (% e)	24.5	29.5	24.6	23.8
Protein (g)	100	128	117	115
Protein (%e)	18.3	16.0	16.7	16.6
Carbohydrates (g)	321	467	429	429
Carbohydrates (%e)	62.1	58.8	61.4	61.9
Net carbs (g)	276	400	358	353
Fiber (g)	45	64	66	71
Trans fat (g)	0.0	0.3	0.2	0.1
Trans fat (%e)	0.0	0.1	0.0	0.0
Sat fat (g)	16.4	25.5	18.9	18.4
Sat fat (%e)	5.8	7.2	6.1	6.0
Sugars (g)	118	180	174	173
Cholesterol (mg)	106	231	175	278
Sodium (mg)	4113	5346	3967	3914
**Energy Expenditure/day**	**Baseline**	**Post-PNP**	**2-Month Follow-up**	**4-Month Follow-up**
Running distance (km)	12.1	12.1	12.0	12.1
Duration (min)	90	79	79	67
Energy expenditure	1045	984	937	875

Data are reported are mean values. Abbreviations: %e—percent energy. Baseline dietary and exercise data was collected for one week prior to start of the PNP, post-PNP data is an average of one-month data collected every day during one month of the program, two-month follow-up is average of two months data collected every day post-program, and four-month follow-up is average of two months data collected every day post-two-month follow-up. Dietary and exercise data from 4-month follow-up through 16-month follow-up was not available.
